# Contrast enhanced MRA with CAPR (Cartesian Acquisition with Projection like Reconstruction) technique - review of initial clinical experience

**DOI:** 10.1186/1532-429X-16-S1-O77

**Published:** 2014-01-16

**Authors:** Manoharan Muthuvelu, Philip M Young, Paul T Weavers, Stephen J Riederer

**Affiliations:** 1 Radiology, Mayo Clinic, Rochester, Minnesota, USA; 2 MR Research Laoratory, Mayo Clinic, Rochester, Minnesota, USA

## Background

Contrast enhanced MRA is a well validated and widely used technique for noninvasive vascular imaging. Significant advances in coil development, parallel imaging, and view-sharing techniques can enable imaging with very high spatial and/or temporal resolution which can be tailored to very specific or problematic clinical situations. Cartesian acquisition with projection like reconstruction (CAPR) is a specific technique developed by our MR Research laboratory which supports very high acceleration factors and has been adopted into clinical practice for a number of clinical scenarios. We will present a brief introduction to the technique and an illustrative review of our early clinical experience using CAPR for clinical imaging.

## Methods

CAPR apportions the Cartesian phase-encoded kY -kZ plane into a low-spatial-frequency center region and a high-spatial-frequency outer annulus. The annulus is further divided into sets of projection-like vanes. The corners of the kY -kZ plane are not sampled and are zero-filled, and data for the unsampled gaps between vanes are estimated by 2D homodyne processing. An individual image update consists of elliptical-centric sampling of the center region and one annular vane set, with view sharing applied from previous samples of the other vane sets. This vane pattern gives a visual appearance similar to projection reconstruction. Alternate vanes are sampled or not sampled, allowing for 2D homodyne detection to be performed across the kY-kZ plane. Because homodyne detection is performed in this plane, full echoes are sampled along the readout (kX) direction.

## Results

We have used this technique in both single station and multi-station implementations, the latter using a novel technique of "fluoro-tracking" enabled by real-time reconstruction of image data to allow interactive decision making by the technologist to track the passage of a contrast bolus through multiple stations. We will present a number of cases illustrating the use, including imaging of arteriovenous malformations, imaging peripheral vascular runoff to facilitate bypass graft planning the setting of failed prior CTA, and imaging of the small vessels of the hands and feet.

## Conclusions

CAPR is a novel and developing MRA technique which can exploit very high spatial and temporal resolution, and early implementation in our practice has shown promising results.

## Funding

None.

